# Understanding the Impact of a Cleft Camp in Aotearoa New Zealand on Sociability, Self-Esteem, and Confidence

**DOI:** 10.1177/10556656251329297

**Published:** 2025-03-28

**Authors:** Kenny Ardouin, Nicola Stock, Tika Ormond, Phoebe Macrae

**Affiliations:** 1 School of Psychology, Speech & Hearing, 2496 University of Canterbury, Christchurch, New Zealand; 2 Centre for Appearance Research, University of the West of England, Bristol, UK

**Keywords:** peers, psychosocial adjustment, quality of life, social support, young adults

## Abstract

**Objective:**

Although camps have been offered previously for cleft and craniofacial conditions, few have been evaluated empirically. This study sought to determine whether a community-led camp peer support event led to improved sociability and self-esteem among camp attendees.

**Design:**

A mixed methods study was employed. Standardized measures were administered prior to camp, immediately after camp, and 2 weeks after returning home from camp. Qualitative data were collected using focus groups on the final day of camp.

**Setting:**

A nationwide 3-day camp in Auckland, New Zealand, organized in partnership between the University of Canterbury and Cleft New Zealand.

**Participants:**

Twenty-seven people with cleft aged 10 to 18 attended the camp, of whom, 22 participated in the research study.

**Main Outcome Measures:**

The Rosenburg Self-Esteem Scale, Harter Sociability Scale, and Body Image Life Disengagement Questionnaire were analyzed using descriptive statistics and *t* tests to compare data across timepoints and to normative data. Focus group data were analyzed using conventional content analysis.

**Results:**

Many camp participants were already well-adjusted prior to camp and therefore did not demonstrate improvements in quantitative measures over time. Contrastingly, those who scored below the norm at baseline demonstrated significant gains in self-esteem and sociability. Focus group data demonstrated that people felt less isolated, more confident, and more knowledgeable about cleft after attending camp.

**Conclusions:**

Residential camps for youth with cleft offer a valuable peer support experience and can result in measurable benefits to young people's sociability and self-esteem, alongside qualitatively reported gains. Offering cleft and craniofacial camps as part of a comprehensive youth program is indicated.

## Background

Being born with a cleft lip and/or palate (cleft) can affect self-esteem and sociability later in life.^1–5^ In turn, low self-esteem and difficult social interactions can impact overall life engagement and be a risk factor for poor mental health.^6–9^ Previous research has shown that high-quality friendships can offer a buffer against negative social experiences such as bullying,^10–12^ and that having a best friend^13^ or multiple friends^14–16^ is important for building resilience. Engaging in peer support may therefore help people to adjust to living with a health condition^17–19^ and improve psychosocial well-being.^20^

### Peer Support

Peer support refers to the opportunity to connect and share knowledge with people with similar life experiences.^17^ Peer support may be delivered online, in person, or over the telephone, in a one-on-one or group-based format, and on an informal or formal basis.^17,18^ Peer support is recognized as an effective mechanism to reach people who may no longer be formally engaged with the health system.^21^ Within craniofacial support services, caregivers are often the beneficiaries of peer support,^22,23^ yet as children become older, the need for peer and community connectedness becomes increasingly important to their own well-being.^24,25^ Targeted peer support interventions for young people with chronic or rare conditions can be effective at reducing social isolation and improving emotional well-being,^26,27^ and in some cases, holistic health outcomes too.^19,28^

Several peer support-based interventions aimed at adolescents and adults with craniofacial conditions have been shown to be effective. From 2011 to 2018, the Cleft Lip and Palate Association (CLAPA) partnered with the Centre for Appearance Research (CAR), to develop and evaluate a cleft-specific peer support service in the United Kingdom which resulted in participants feeling more confident, connected, and resilient.^29^ Cleft Lip and Palate Association and CAR partnered again from 2018 to 2021 to deliver the Adult Services Project, which provided opportunities for adults with cleft to connect with the community and access peer support through annual conferences and a podcast.^30^ Researchers in the United States evaluated a support conference for people with Moebius syndrome and found that adults benefitted socially from meeting others and reported reduced stigma and increased knowledge about their condition after attending.^22^ Each of these studies demonstrated that peer support interventions offer a valuable adjunct to medical care.

A key example of peer support that has shown promise in young people with cleft is a multiday camp (sometimes referred to as a “residential weekend”).^29^ Camps typically involve a program of activities designed to foster cooperation and teamwork and develop self-esteem within attendees and provide an opportunity for people to build connections with like-minded people outside of their existing support and family network.^31^ Cleft camps globally have been run by a variety of different personnel, including cleft team clinicians, volunteers, other members of the cleft community, and/or staff from national support groups/charities.^32^

One such camp was run in tandem by a Canadian paediatric hospital and the national support group, About Face.^32^ The camp consisted of structured teambuilding activities such as rock climbing and ropes courses, designed to offer the opportunity for spontaneous conversation between attendees and camp leaders about facial differences. Data from qualitative focus groups demonstrated that participants enjoyed and appreciated participating in the camp. Using unvalidated postcamp Likert scales, participants also self-reported reduced feelings of isolation and feeling better about themselves after the camp. However, the study found that little spontaneous discussion occurred and concluded that formal facilitator-led dialogue may have been beneficial.^32^ A US-based study investigated the outcomes of 3 craniofacial camps hosted by Seattle Children's Hospital over a 2-year period. Data collected through telephone interviews with 105 participants aged 7 to 16 years found that spending time with peers and making new friends were the highlights of the camp experience.^33^ Finally, a 5-day residential camp hosted in the United States for 31 children aged 9 to 18 years with various craniofacial conditions^34^ used the Rosenberg Self-Esteem Scale (R-SES) to collect standardized data at multiple timepoints (precamp, immediate post, 6-8 weeks post).^35^ The results showed significant gains in self-esteem at the end of the camp. However, these gains diminished somewhat 6 to 8 weeks later, although they remained higher than the precamp baseline.

### Use of Camps as a Peer Support Intervention in Broader Health Conditions

Camps have also been successfully implemented within the broader health field. A study of 77 people aged 7 to 19 years with burn injuries who attended 1 of 4 camps over a 4-year period found that participants self-reported increased confidence on an open-ended questionnaire after attending the camps. However, the study did not find statistically significant changes in the standardized measures used (Social Competence with Peers Questionnaire, Strength and Difficulties Questionnaire and Self-Perception Profile for Children).^36^ Furthermore, those involved in running the program expressed frustration that the positive effects they had observed were difficult to quantify and were not fully reflected in the measures used to report study results.^36^

Diabetes New Zealand routinely delivers camps for 8 to 12-year-olds living with type 1 diabetes. These camps include the whole family, to provide parents and siblings the opportunity to meet other families. Although these camps have not been evaluated empirically, the camp coordinator reported observed benefits including the relationships developed between the youth themselves, and with the medical staff who they would normally only see at clinics.^37^

The positive influence of camps on social skills and self-esteem in the short term has also been found among children with cancer.^38^ Notably, this systematic review of 15 camps only found these gains when children attended camp without their parents. Limitations identified within the review included the lack of use of standardized measures and the failure to include a comparison group.

### Gaps in Current Knowledge

While the findings of existing studies are encouraging, the evidence for a camp's efficacy is often anecdotal, measured quantitatively at a single timepoint, or measured using unvalidated measures without a comparative reference group. First, the collection of pre- and postcamp data to understand change over time is important. In particular, an understanding of whether any improvements from camp were sustained after participants returned home would offer greater insight into the translation of effects into everyday life. Standardized measures should be used wherever possible to ensure a robust design, supplemented with rich qualitative data to capture and explore the more nuanced findings of camp interventions. The collection of mixed methods data may avoid the outcome where quantitative data do not capture all the effects of attending camp as described in previous research.^36^ Young people should also be given the opportunity to participate in camp without their parent(s) being present to maximize interactions with their peers. Providing empirical evidence that camps offer tangible benefits to children and adolescents affected by craniofacial conditions would help facilitate the inclusion of these into the care pathway, prioritize their delivery, and reduce funding barriers.

### Aims and Hypotheses

The aim of the current study was to evaluate a community-based cleft camp in New Zealand, with the goal of assessing the extent to which participating in a camp program with cleft-affected peers influenced participants’ self-reported sociability, life engagement, and self-esteem.

Based on existing literature, it was hypothesized that participants would report increased levels of sociability, life engagement, and self-esteem immediately following the camp. It was also hypothesized that these scores would drop at 2 weeks postintervention yet remains in line with or higher than the initial baseline. It was anticipated that the collection of qualitative data would add further insight into any effects of the camp.

## Methods

### Design

This study utilized an empirical mixed methods design. Standardized measures were used to assess sociability, life engagement, and self-esteem at 3 time points (baseline, immediately postcamp, follow-up). Structured focus groups with 4 to 6 participants in each were held on the final day of camp and were audiorecorded and transcribed verbatim.

### Ethics

Ethical approval for this study was granted by the University of Canterbury Human Research Ethics Committee in May 2023 under approval number 2022-121.

### Consultation With Māori

As the project aimed to involve Māori participants and the findings were anticipated to have implications for the Māori cleft community, Māori consultation was undertaken and approved by the Ngāi Tahu Consultation and Engagement Group. For this study, consultation involved a written submission of the research project followed by a face-to-face meeting (hui) with their delegated representative(s). In this hui, the research proposal is discussed to ensure that the research is conducted in a manner which is sensitive to Māori interests and is culturally appropriate. This includes reviewing all supporting documentation and recruitment strategies to ensure all participant engagement supports Māori participation. For example, within this study, Māori consultation identified the importance of providing ground transport (as well as air transport) to collect participants from their neighborhood to avoid inequities arising from living away from a major city.

### Eligibility and Recruitment

Individuals were eligible to attend camp if they lived (anywhere) in New Zealand, were aged between 10 and 18 years at the time of the camp, and were born with any type of cleft (including cleft arising as part of a syndrome). Attendees also had to commit to attending the entire 3-day, 2-night camp and deem themselves medically well enough to participate in camp. All camp attendees were eligible to participate in the research study.

In addition to completing the mandatory camp registration, camp attendees were provided with information about the research. If they wished to participate in the study, they and/or their parent/guardian were asked to complete an assent (<18 years) or consent (18 years+) form. Participation in the research was not required for people to be able to attend the camp.

People aged 20 or over who had been born with cleft were eligible to apply to attend camp as a camp leader (volunteer). Given their privileged role within the development of the camp program, volunteers were not eligible to participate in the research study.

### Camp Overview

The 3-day camp took place in a beach suburb within Auckland, New Zealand, in April 2024. The camp was developed according to prior literature and in collaboration with national charity, Cleft New Zealand. The program contained physical activities (eg, rock climbing and kayaking), teamwork and problem-solving activities (eg, raft building), whole-group activities (eg, movie night), a talk from a guest speaker with lived experience of cleft, and a Q&A session about cleft, in addition to scheduled free time, and mealtimes.

Participants and staff were accommodated in motel-style accommodation with a maximum of 4 people per room. Participants were age and gender-matched to ensure that they were lodging with people of a similar age and the same gender. For many activities, participants were split into 2 groups based on age: 10 to 12 years and 13 to 18 years. These 2 age groups were selected to reflect those who were still attending primary school and those who were at high school. There were 27 camp attendees and 11 camp leaders, and a minimum ratio of 1 volunteer to 4 camp attendees was upheld at all times. Volunteers were recruited following rigorous selection criteria, including mandatory police vetting through NZ Police and child safeguarding training. The camp volunteers were provided with comprehensive training which followed the guidance of CLAPA's residential weekend volunteer training program.^39^ This program was adapted for the local camp program, while child safeguarding training consisting of a series of e-learning modules created by the New South Wales Office of the Children's Guardian.^40^

Participants and volunteers traveled across New Zealand to participate in camp. All transport (air and road) and camp costs (accommodation, meals, activities) were covered by the project grant to reduce barriers to attendance for attendees and volunteers. Attendees were asked to pay a 100 NZD deposit (∼USD $60) to demonstrate their commitment to attend camp. This deposit was fully refundable after they had attended camp. The deposit requirement was waived on a case-by-case basis for people in financial hardship.

### Quantitative Outcome Measures

Measures of self-esteem, sociability, friendships, and life engagement were administered to participants at 3 time points: (1) within the 2 weeks leading up to the camp (a precamp baseline measure), (2) on the final day of camp (an immediate postcamp measure), and (3) 2 weeks following the camp (a follow-up measure). These timepoints were based on the timings used in a similar recent study^41^ and were intended to facilitate data collection at periods of maximal engagement to reduce attrition. Participants completed the pre- and follow-up measures at home using an online Qualtrics survey and completed the immediate postmeasure at camp using printed copies of the Qualtrics survey.

Measures were selected on the basis that they were standardized, validated, and would offer a comparison to other populations. Measures that had been used in similar studies were favored where the previous criteria were met. The study measures included:

- *The R-SES*^35^: The R-SES consists of 10 questions, each measured on a 4-point scale from “strongly agree” to “strongly disagree.” Scores range from 0 to 30, with scores between 15 and 25 considered to be within a normal range and scores below 15 indicating low self-esteem.^35^ The scale is psychometrically sound, widely used globally, can be administered to young people, and has been used to evaluate self-esteem in similar studies.^34^
- *Norwegian Close Friends Scale*^42^ *and Harter Sociability Scale (SPP-Ad)*^43^: The Norwegian Close Friends Scale provides a measure of participants’ self-perception of the number and quality of their friendships, while the Harter Sociability Scale is taken from the larger Self-Perception Profile for Adults and offers additional insights into individuals’ behavior in the presence of others. The Norwegian Close Friends Scale consists of 2 questions on a 4-point scale, with higher scores indicating greater satisfaction with friendships. The Harter Sociability subscale consists of 4 questions on a 4-point scale (consisting of “describes me very poorly,” “describes me quite poorly,” “describes me quite well,” and “describes me very well”). The Norwegian version of the scale was used, which is considered more straightforward to complete than the original and replicates the same factorial pattern, yet has been shown to achieve better reliability and convergent validity.^44^ Scores on the SPP-Ad range from 1 to 4, with higher scores indicating greater sociability. Both measures have been used previously with existing New Zealand^45^ and United Kingdom^46^ adult cleft samples which guided their selection for this study.
- *Body Image Life Disengagement Questionnaire (BILD-Q)*^47^: This scenario-based measure includes 10 questions about specific situations where body image concerns may impact people's engagement in daily activities (eg, taking public transport). Each of the 10 questions is rated on a 4-point scale from “Hasn’t stopped me at all” to “Stopped me all the time.” Scores on the BILD-Q range from 1 to 4 with higher scores indicating greater levels of life disengagement.

To preserve anonymity for research participants while enabling the research team to evaluate change over time, participants were asked to generate a participant code and to use this same code each time they completed the survey.

### Qualitative Focus Groups

To further understand the experience of people who attended camp, structured focus groups were run on the final day of camp. Participants split themselves into 5 groups of 4 to 6 participants. A camp volunteer audiorecorded the group's discussion and transcribed key points on paper during the session. Camp attendees who did not consent to participate in the research played games in a separate part of the venue with a camp volunteer.

The focus groups consisted of a series of prompt questions to encourage participants to reflect on their camp experiences (Table 1). The 5 focus groups were run concurrently and lasted for 90 min. The focus group questions were informed by previous published^29^ and unpublished work, and the clinical and research expertise of the research team.

**Table 1.** Camp Evaluation Focus Group Prompt Questions.

Group questions	
Question #	Question
1	What activity or aspects of camp did you most enjoy and why?
2	How did you feel about coming to camp before you got here?
3	How do you feel about camp now that you have experienced it for yourself?
4	Were there any activities or aspects of camp that you didn’t like, or that you couldn’t do? Why?
5	If you could change one thing about the camp, what would it be and why?
6	How do you feel about your cleft after having come to camp?
7	What is one new thing that you learned from being on camp?
8	Would you come to another cleft camp in the future? Why or why not?
9	What would you say to another young person who hasn’t been to cleft camp before to encourage them to come?
10	What is the most important thing that you have got out of the cleft camp?
11	What is your favorite memory that you are taking home with you?
12	What (if anything) will you do differently or think differently about once you go home as a result of camp?

Continuum “Post-It note” questions	
1	Meeting other people with cleft makes me realize that I’m not alone
2	I feel a bit more confident about myself after coming to camp
3	I feel a bit more positive about myself after coming to camp

Toward the end of the focus group, all the groups were brought back together. Each participant was given 3 colored post-it flags and was asked to place a flag on a continuum ranging from “disagree a lot” to “agree a lot” in relation to 3 questions.

### Data Analysis

For each of the quantitative measures, descriptive statistics (means and standard deviations) were calculated for the whole cohort. The Norwegian Close Friends Scale data were reported descriptively alongside comparative United Kingdom and New Zealand cleft cohort data to examine any similarities or differences between samples.

One-way repeated-measures analysis of variance (ANOVA) were carried out on the Harter SPP-Ad, BILD-Q, and R-SES data using IBM SPSS software (version 29.0.0.0 (241)) to compare the cohort's scores over time: at baseline, immediately after camp, and 2-week post-camp. This was conducted following a previously published procedure for analyzing repeated measurement data in clinical trials where Mauchly's test of sphericity is conducted first, followed by a test of within-subject effects across timepoints.^48^ If any of the within-subject effects are statistically significant, Bonferroni pairwise comparisons were used to establish which comparisons demonstrated differences.^48^ Additionally, independent samples *t* tests were carried out using GraphPad software to compare the cohort data to the published general population normative data at precamp baseline and the 2-week post camp timepoint.

Additional post hoc quantitative analyses were carried out where the sample was stratified according to baseline scores (ie, above or below the general population normative mean) for each of the SPP-Ad, BILD-Q, and R-SES to identify whether baseline score influenced the degree of change over time. All the aforementioned a-priori analyses were rerun for each of these above- and below-norm groups for the 3 measures, resulting in rerunning the a-priori analyses 6 additional times.

Data from the focus groups were transcribed using Otter.AI software, and the transcriptions were checked by the lead author for accuracy. The transcripts were then analyzed inductively using conventional content analysis.^49,50^ Content analysis was initially performed on the answers to each question to identify initial codes and exemplar quotes. The first and second authors then discussed and refined the initial codes, merging similar responses across different questions together and attributing frequency counts to each code. Data from the 3 continuum questions were presented descriptively. Key findings from the focus group data were also summarized narratively.

## Results

### Participants

From a total of 27 eligible camp attendees invited to participate, 24 consented to participate in the research study (12 male, 12 female). The mean age of participants was 11.93 years. Fifteen participants came from the North Island (62.5%) and 9 from the South Island (37.5%). The most recent census showed 75% of New Zealand's population resides in the North Island.^51^ The sample included both Māori and non-Māori.

### Changes in Outcomes Over Time

All 24 participants completed the precamp and immediately post camp quantitative questionnaires. Two participants were lost to follow-up and therefore 22 full responses were included in the analysis. Repeated measures ANOVA data for the SPP-Ad, BILD-Q and R-SES are presented in Table 2. Tests of within-subject effects across timepoints found no significant differences between the timepoints, except for the above general population norm SPP-Ad group (n* *= 11), which showed a decrease in sociability between the precamp baseline and end of camp, and an increase in sociability from the end of camp to postcamp follow-up. The effect size of differences over time within this group is considered large (η² = 0.44). Pairwise comparisons were then reported for this group (within Table 2).

**Table 2.** Repeated Measures ANOVA Across Measured Timepoints.

Harter Sociability Scale (SPP-Ad)							
Results of Mauchly's test of sphericity (by group)							
Within-subjects effect	Mauchly's W	Approx. chi-square	*df*	*P* value (sig.)	Greenhouse-Geisser	Huynh-Feldt	Lower bound
Whole cohort (n = 22)	0.86	2.95	2.00	.229	0.88	0.95	0.50
Below gen pop norm (n = 11)	0.90	0.98	2.00	.613	0.91	1.00	0.50
Above gen pop norm (n = 11)	0.48	6.64	2.00	.036^a^	0.66	0.72	0.50

Test of within-subject effects across timepoints (by group)							
Source		Type III sum of squares	*df*	Mean square	*F* value	*P* value (sig.)	Partial eta squared
Whole cohort (n = 22)	Sphericity assumed	0.59	2.00	0.29	2.14	.131	0.09
Below gen pop norm (n = 11)	Sphericity assumed	0.39	2.00	0.20	1.65	.217	0.14
Above gen pop norm (n = 11)	Greenhouse-Geisser^b^	1.56	1.31	1.19	7.72	.011^a^	0.44

Bonferroni Pairwise Comparisons (Above gen pop norm; n = 11)							
(I) Time	(J) Time	Mean difference (I-J)	Std. error	*P* value (sig.)	95% confidence interval for difference (lower bound)	95% confidence interval for difference (upper bound)	
Precamp	End of camp	0.50	0.14	.006^a^	0.18	0.82	
(Baseline)	Postcamp	0.09	0.08	.267	−0.08	0.26	
End of camp	Postcamp	−0.41	0.17	.036^a^	−0.79	−0.03	

Rosenberg Self-Esteem Scale (R-SES)							
Results of Mauchly's test of sphericity (by group)							
Within-subjects effect	Mauchly's W	Approx. chi-square	*df*	*P* value (sig.)	Greenhouse-Geisser	Huynh-Feldt	Lower bound
Whole cohort (n = 22)	0.81	4.13	2.00	.137	0.84	0.91	0.50
Below gen pop norm (n = 9)	0.94	0.47	2.00	.789	0.94	1.00	0.50
Above gen pop norm (n = 13)	0.29	13.80	2.00	.001^a^	0.58	0.61	0.50

Test of within-subject effects across timepoints (by group)							
Source		Type III sum of squares	*df*	Mean square	*F* value	*P* value (sig.)	Partial Eta Squared
Whole cohort (n = 22)	Sphericity assumed	12.39	2.00	6.20	0.68	.512	0.03
Below gen pop norm (n = 9)	Sphericity assumed	6.22	2.00	3.11	0.26	.777	0.03
Above gen pop norm (n = 13)	Greenhouse-Geisser^b^	8.97	1.17	7.70	0.58	.483	0.05

Body Image Life Disengagement Questionnaire (BILD-Q)							
Results of Mauchly's test of sphericity (by group)							
Within-subjects effect	Mauchly's W	Approx. chi-square	*df*	*P* value (sig.)	Greenhouse-Geisser	Huynh-Feldt	Lower bound
Whole cohort (n = 22)	0.25	27.53	2.00	≤0.001^a^	0.57	0.58	0.50
Below gen pop norm (n = 4)	0.04	6.49	2.00	.039^a^	0.51	0.53	0.50
Above gen pop norm (n = 18)	1.00	0.00	2.00	.999	1.00	1.00	0.50

Test of within-subject effects across timepoints (by group)							
Source		Type III sum of squares	*df*	Mean square	*F* value	*P* value (sig.)	Partial eta squared
Whole cohort (n = 22)	Greenhouse-Geisser^b^	0.16	1.14	0.14	1.19	.295	0.05
Below gen pop norm (n = 4)	Greenhouse-Geisser^b^	0.59	1.02	0.57	0.89	.416	0.23
Above gen pop norm (n = 18)	Sphericity assumed	0.30	2.00	0.02	1.58	.221	0.09

Abbreviation: ANOVA, analysis of variance.

^a^ Finding is statistically significant (*P* < .05).

^b^ The Greenhouse-Geisser measure is indicated in lieu of Sphericity assumed when the result of the corresponding Mauchly's Test of sphericity is statistically significant.^48^

The Norwegian Friendship Scale data are presented using descriptive statistics to compare to other cohort data (Table 3). Comparison of this study's pre- and postcamp means on the SPP-Ad, R-SES, and BILD-Q with published normative data is also presented in Table 3.

**Table 3.** Comparison of Measures to Normative or Cohort Data Pre- and Postcamp.

Norwegian Close Friends Scale					
	Precamp timepoint (n = 22)	End of camp timepoint (n = 22)	Postcamp timepoint (n = 22)	NZ Cleft Adult Cohort Data^45^ (n = 17)	UK Cleft Adult Cohort Data^46^ (n = 176)
Number of people with 4+ friends	n = 12/22 (54.5%)	n = 17/22 (77.3%)	n = 12/22 (54.5%)	n = 14/17 (82.4%)	n = 106/176 (60.2%)
Rated friendships as “excellent”	n = 12/22 (54.5%)	n = 16/22 (72.7%)	n = 15/22 (68.2%)	n = 13/17 (76.5%)	n = 87/176 (49.9%)
Rated friendships as “good”	n = 10/22 (45.5%)	n = 5/22 (22.7%)	n = 7/22 (31.8%)	n = 4/17 (23.5%)	n = 68/176 (38.6%)
Rated friendships as “fair”	n = 0/22 (0.00%)	n = 1/22 (4.54%)	n = 0/22 (0.00%)	n = 0/17 (0.00%)	n = 21/176 (11.93%)
Harter Sociability Scale (SPP-Ad)					
	General population norm (n = 141)	Precamp study data	*T* test general population to precamp	Postcamp study data	*T* test general population to postcamp
Whole cohort—Mean (SD) (n = 22)	3.19 (0.61)	3.07 (0.40)	*P *= *** ***.374	3.15 (0.43)	*P *= .768
Participants below gen pop norm—Mean (SD) (n = 11)	3.19 (0.61)	2.75 (0.22)	*P *= .019^a^	3.00 (0.46)	*P *= .314
Participants above gen pop norm—Mean (SD) (n = 11)	3.19 (0.61)	3.39 (0.26)	*P *= .283	3.30 (0.35)	*P *= .557
Rosenburg Self-Esteem Scale (SES)					
	General population norm (n = 503)	Precamp study data	*T* test general population to precamp	Postcamp study data	*T* test general population to post camp
Whole cohort—Mean (SD) (n = 22)	22.62 (5.80)	22.91 (5.42)	*P *=* *.818	23.59 (5.67)	*P *= .443
Participants below gen pop norm—Mean (SD) (n = 9)	22.62 (5.80)	17.89 (4.34)	*P *=* *.015^a^	19.00 (4.95)	*P *= .063
Participants above gen pop norm—Mean (SD) (n = 13)	22.62 (5.80)	26.39 (2.60)	*P *= .020^a^	26.77 (3.63)	*P *= .011^a^
Body Image Life Disengagement Questionnaire (BILD-Q)					
	General population norm (n* *= 2034)	Precamp study data	*T* test general population to precamp	Postcamp study data	*T* test general population to post camp
Whole cohort—Mean (SD) (n = 22)	1.41 (0.54)	1.20 (0.30)	*P *=* *.986	1.31 (0.57)	*P *= .388
Participants below gen pop norm—Mean (SD) (n = 4)	1.41 (0.54)	1.78 (0.17)	*P *= .171	2.15 (0.96)	*P *= .006^a^
Participants above gen pop norm—Mean (SD) (n = 18)	1.41 (0.54)	1.07 (0.09)	*P *=* *.008^a^	1.12 (0.18)	*P *= .023^a^

^a^ Finding is statistically significant (*P* < .05).

#### Friendships

At baseline (precamp), participants reported having a similar number of friends to those reported in a UK Cleft Adult Cohort,^46^ however, had fewer friends than those in a New Zealand Cleft Adult Cohort.^45^ At the end of camp, the proportion of participants reporting having 4 or more friends had increased to levels consistent with the New Zealand Cleft Adult Cohort, however subsequently, it declined back to baseline levels 2-week postcamp, indicating no sustained improvement in number of friends. Similarly, at baseline, the proportion of participants who rated their friendships as “excellent” or “good” was similar to those of the UK cohort; however, following the camp, there was an increase of 20% to 25% in the number of participants reporting their friendships to be “excellent.” This increase was sustained 2-week postcamp, indicating an improvement in self-perceived friendship quality following the camp experience.

#### Sociability

Half of participants (n = 11) scored in line with general population norms, while half reported scores below the norm. For the group who scored less favorably regarding sociability at baseline, there were no statistically significant within-subject effects across timepoints. However, in contrast to their baseline, the follow-up score was no longer significantly different to the general population norm, indicating this group was in line with general population norms by the end of camp.

For the group who scored more favorably regarding sociability at baseline, a repeated measure ANOVA with a Greenhouse-Geisser correction determined there was statistically significant change in the mean sociability score (*F *= 7.72, *P *= .01). Bonferroni pairwise comparisons revealed a slight decrease in sociability at the end of camp (−0.50 ± 0.14, *P *< .01), followed by a subsequent slight increase in sociability between the end of camp and postcamp follow up (0.41 ± 0.17, *P *<* *.05).

#### Self-esteem

Repeated measures ANOVA found no within-subject effects across timepoints for any group on the R-SES measure. When compared to normative data, most participants scored in line with or higher than general population norms (n* *= 13), while 9 participants reported scores below the norm. For the group who scored less favorably regarding self-esteem at baseline, as with sociability, the follow-up score was no longer significantly different from the general population norm, indicating this group was in alignment with general population norms by the end of camp. For participants who reported a more favorable score in self-esteem at baseline, they continued to do so at follow-up with the group remaining significantly above the general population norm (*t(df) *= 2.56(514), *P *< .05).

#### Life engagement

Most participants scored in line with or higher than general population norms (n = 18), while 4 participants reported scores below the norm. No statistically significant within-subject effects in life engagement over time were observed for any group. However, when compared to general population norms, there was a significant change between the group that was below the general population norm and the follow-up timepoint (*t(df) *= 2.73(2036), *P *< .01), indicating they had a high degree of BILD relative to the norm. This is likely influenced by one participant's score on the BILD-Q at follow up which was particularly poor.

### Qualitative Data

Insights from the focus groups generated 3 overarching categories: participant sentiments before camp, participant experiences during camp, and participants’ postcamp reflections. The latter category is also divided into 4 subcategories: experience of meeting others with cleft, personal development, peer support and emotional well-being, and sentiment toward the camp experience. These categories (and subcategories), their codes, frequency counts, and exemplar quotes are presented in Table 4.

**Table 4.** Focus Group Content Analysis Categories.

Category 1: Participant sentiment before camp		
Code	Frequency	Exemplar quote(s)
Nervous/anxious	18	“I was nervous about meeting new people.” “I was afraid no-one would like me.” “I was worried and scared about my voice.” “I was nervous there wouldn’t be anyone my age.” “I was scared to be left alone [without my parents].”
Excited	15	“I looked forward to meeting people like me.” “I was so excited to relate to people with different types of cleft and make new friends.”
Ambivalent	3	“I wasn’t sure how I felt.” “My parents wanted me to come.” “I didn’t really think about it beforehand.”
Category 2: Participant experiences during camp		
Code	Frequency	Exemplar quote(s)
I enjoyed the activities	36	“All the activities were quality!” “I loved bowling people over in the bubble soccer!” “I really enjoyed the climbing—I went so high!” “Kayaking—I was super good at it and got to go so deep!” “Raft building, and getting on top of the raft, and then everyone else getting on top of the raft, and then someone fell into the water!”
Ideas for future camp activities	11	“Add prizes for activities—e.g., best-dressed movie night, best organized etc.” “Add a sports tournament” “Add an obstacle course” “Let's add surfing!”
More opportunities to participate in smaller groups	5	“I’d prefer it if there were fewer people.” “Same number of people, smaller groups.” “It's easy to get lost in the crowd.”
Altering the timings of the camp program	4	“Stretch things out so we’re not so tired.” “Have longer activity times.” “More craft/free time activities.” “More free time.”
Feeling overwhelmed	3	“I didn’t like some people.”
Friendly staff	2	“Keep the camp leaders the same!”

Category 3: Postcamp reflections		
Code	Frequency	Exemplar quote(s)
*Subcategory 1: Experience of meeting others with cleft*		
Feeling less isolated because of camp	32	“Just how important representation is and having a place where you can see yourself and meet other people like you is so important for all of us to have.” “You can relate to people here.” “Interesting to meet people who looked like me and have similar experiences.” “I feel better now I know I’m not alone.” “It's the first time I’ve met people like me.” “Cool to be able to meet people and not have to explain everything about cleft”
Made new friends	31	“I got to know new people.” “I made new friends!” “Making new friends and sharing new experiences with them, making new memories.” “Meeting new friends and talking with them, particularly at night.”
Sharing my cleft experience with others	8	“Being able to tell people things that I wish I had known before my treatment.” “Meeting people with the same or similar experiences and sharing how we felt.” “Getting to talk with everyone.” “Talking with people.”
Appreciation of others cleft experiences	7	“A great understanding of people's experiences.” “Seeing the expression on someone's face whenever someone mentioned an experience that they’d had and said ‘me too’.” “When Sam mentioned one of his surgeries…I saw three of the girls turn around, look at each other and go “I’ve had that one too!”…and that has happened lots and lots of times while we’ve been on this camp.” “Almost anything that we said to each other we were like ‘oh same!’ to each other.”
I want to connect with more people with cleft	6	“I’d love to meet new people coming in.” “Meeting new cleft kids would be amazing.”
Desire to be actively involved with the cleft community	4	“I liked meeting some old friends who I knew when I was younger.” “Make sure I’m present on the Cleft NZ Facebook page” “I’ll be thinking that I’m part of the community”
*Subcategory 2: Personal development*		
Gaining confidence	11	“More confident because cleft was my one ‘insecurity’ and now I don’t feel so bad about it.” “The voice is the thing I’m most insecure about. Coming here, everyone talked like me—I was so excited.” “I don’t need to be afraid to be who I am.” “My cleft is something that I can be proud of and that other people can be proud of because it's not a bad thing, it's a really good thing.” “Being more proud of my cleft, not covering it up or being ashamed of it.”
Feel better informed about cleft	11	“I learnt a lot of things I didn’t know.” “I feel so much better there's a whole range of cleft types—to see we were all similar and went through similar experiences.” “I learnt that it can be genetic.” “1/700 people get cleft = lots of people!” “I learnt more info about future surgeries and recovery periods.” “It still counts as a cleft if it's been repaired.”
Pushing my own boundaries	10	“I put myself out of my comfort zone.” “Doing things I wouldn’t do otherwise.” “Realising that when I think I can’t do things, I actually can.” “I enjoyed rock climbing because it was something I never thought I would see myself doing.”
*Subcategory 3: Peer support and emotional well-being*		
Feeling better after camp	5	“Camp made me feel better about my cleft.” “I feel better.”
Optimism for the future	5	“I feel fine because it doesn’t affect your future career.” “Having a more positive mindset about having a cleft.” “I found a new sense of purpose.”
No change as a result of camp	4	“I think of my cleft the same—as a thing that doesn’t stop me.” “Much the same.”
Connected with emotional awareness/well-being	4	“Everyone's cleft is different and different things affect different people.” “I learned about emotions going into surgeries.” “I learnt more about myself.” “Everyone has their own challenges and it just depends how you look at them for how you’re going to overcome them yourself.”
Wish to provide peer support for others	4	“I would love to come back as a camp leader.” “I will keep trying to advocate for young people with cleft.”
Developing coping strategies	3	“Now I know when people say ‘Why do you talk like that?’ I can say, ‘I have a cleft’.” “Being able to laugh about stuff like surgeries.”
Comparisons with others	3	“I feel lucky because other people still need surgeries for their problems.” “My cleft is bigger than some people's.” “Thinking a bit more about the people around me and taking my eyes off myself a bit.”
*Subcategory 4: Sentiment toward the camp experience*		
Camp was an enjoyable experience	22	“I loved it.” “I was really happy that I went—I really liked it” “I am very pleased I came—I enjoyed every activity!” “Super amazing!”
Want the camp experience to continue	14	“Sad to leave.” “I want to do it again!” “I wanted it to be longer.” “I really hope they’re going to do it again.” “I want to do it 10,000 times!” “I would love to come back.”
Gratitude for camp	3	“Grateful” “Thank you so much for having me here.”
Camp is a supportive environment	2	“Seeing how supportive and caring everybody is.” “Feeling secure.”
Camp exceeded expectations	2	“I thought it would be boring but it wasn’t.” “Better than expected.”
Concerns about reintegrating into the wider community	1	“It's going to be weird when we go back home and no one has a cleft.”
Feelings of surprise	1	“I felt shocked because I didn’t know I would see people like me.”

The qualitative data demonstrate participants held mixed feelings about their pending camp experience, with many commenting they felt nervous, excited, or both. Some participants had not given this a lot of thought or were attending because they felt their parents had wanted them to. During camp, participants were overwhelmingly positive about the camp activities that were offered. Some participants would have preferred to participate in smaller groups, and/or that the timings of activities could be adjusted to allow for more free time or more time to complete each activity. Participants’ reflections of the camp overall indicated they felt less alone after attending camp, and that they had made new friends. Camp was an enjoyable experience that many would participate in again. Furthermore, the camp experience had helped participants to gain confidence, try things they had not tried before or that they previously felt they could not do, and to feel better informed about cleft itself.

**Figure 1.**
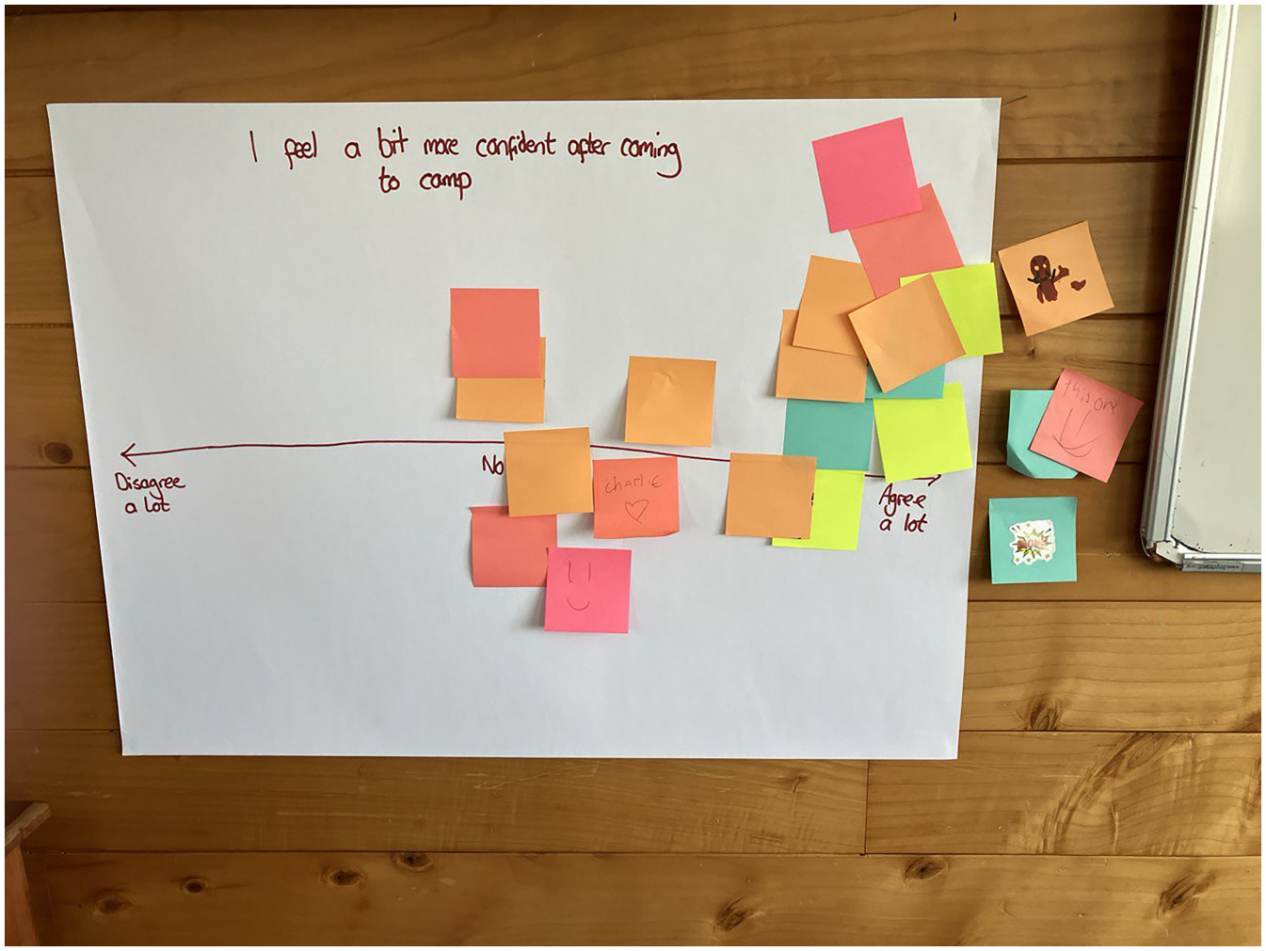
All participants reported that after coming to camp, they either noticed no change in their confidence (center of scale) or that they agree to varying extents that they feel more confident after attending the camp.

The data from the first post-it note continuum (see Figure 1 for an example) showed that 7 participants reported that camp made no change to any feelings of isolation, while 14 indicated that camp helped them feel less isolated. One person indicated that meeting other people with cleft may have made them feel more alone, although no additional context for this is available. On the second post-it note continuum, 7 participants indicated no change in their confidence, while 15 indicated that they felt more confident after attending camp. On the final continuum, 5 participants reported no change in how they felt about themselves, while 17 reported that camp had helped them feel more positive about themselves. No-one reported feeling less confident or positive about themselves after camp. Some post-it notes (included in the counts above) were placed beyond the highest point of the scale for the first and second continuums. These participants told the researcher they “couldn’t agree enough” with these statements.

## Discussion

The aim of this study was to evaluate a community-based cleft camp in Aotearoa New Zealand. When considering the whole cohort, the hypotheses that camp would increase levels of sociability, life engagement, and self-esteem were rejected. However, it was observed that most participants reported baseline scores that were aligned with, or more favorable than general population norms. Given that the whole cohort data rejected the hypotheses, yet most participants were aligned with or above the general population, we conducted post hoc analyses to determine whether the whole cohort findings were true of all participants. Therefore, the data were subsequently stratified according to baseline score, which indicated improvements in self-perceived sociability and self-esteem relative to the norm were evident in the group with less favorable baseline scores. These findings were supported by the qualitative and continuum data, which indicated overall enjoyment of camp but with variability in the level of need. No improvements in overall life engagement were observed in either group.

### Clinical Implications

The findings of this study indicate that a community-based camp intervention for young people with cleft can support, and in some cases increase self-perceptions of friendship quality, sociability, and self-esteem. These findings may be most pronounced for young people reporting scores lower than the general population at baseline. Teams preparing to host a cleft camp with limited resources could therefore decide to offer camp as an intervention only to those who may benefit most by conducting prescreening. However, taken with the qualitative data, there was evidence to suggest that attending camp was a positive experience, even when the participant was already well-adjusted. Furthermore, it is possible that having well-adjusted participants at camp was a positive influence on those who entered the camp with lower baseline scores. The findings of the present study align with evaluations of previous camps for young people with health conditions^32,34,36,38^ and lend further support to the value of peer support interventions in cleft and the wider field.

This study also offers recommendations for organizations wishing to host a camp intervention. First, given the broad appeal of the camp, the segregation of elements of the camp program into 2 age groups, and participants’ reported desire to return in future, it is indicated that camps may be beneficial at different stages of the cleft journey depending on when an individual feels ready to engage. This also would provide multiple opportunities for someone to engage if they felt uncomfortable or anxious about attending at a younger age. Camps should therefore be sustainable and offered at regular frequency to help them to build momentum within the community. Some places such as the United Kingdom offer multiple camps per year,^52^ whereas other organizations may be better placed to offer them annually or biennially. In any case, the cycle should be negotiated with thought to the lead time needed to secure funding, organize a venue, recruit staff and participants, and develop a strong activity program.

There is also clear value in these camps being offered for the relevant community, *by* the community themselves (eg, the cleft community in this case). Several participants in this study discussed the benefits of having people with lived experience as camp leaders and/or expressed a desire to return to camp in future as a camp leader. Partnerships between support organizations, healthcare teams, and research teams to organize, deliver, and evaluate camps could maximize the resources available and the impact of these interventions. Encouraging past attendees to return in this way enables them to remain involved with the community, develop additional skills and responsibilities, and provide new camp attendees with a leadership team who can offer peer mentoring and support. Involving people with lived experience as youth peer supporters has been found to strengthen youth support services.^53^ The development of a broader youth program to support this transition could therefore be of benefit.

This study received grant support to subsidize travel and registration. The average cost per person to attend camp (transport, accommodation, meals, activity costs etc) exceeded NZD 1100 (∼USD $700), which would place camp out of financial reach for many families, including those who may be most vulnerable and who stand to benefit the most from the experience. Accessing funding to provide a subsidized camp experience will ensure that all those who stand to benefit from such an intervention are provided with the necessary resources to attend.

Finally, several participants expressed anxiety prior to attending camp. While it was encouraging to see that everyone who registered for camp did follow through with attendance, it is possible that other people considered attending, but decided it was too anxiety-inducing or overwhelming. To be able to travel away from home for 3 (or more) days requires a level of independence and resilience. It is therefore possible that camp studies represent a subset of the community who are already well-connected, motivated, and have strong family support. The finding that those who may stand to gain the most from an experience such as camp are likely to be the same people who find it too difficult to put themselves forward to attend has previously been documented in a study with the Moebius Syndrome community.^22^ To address this challenge, it could be important to clearly communicate the purpose of the camp to potential attendees and to share case studies of people who have attended previously, to reduce anxiety and increase engagement. Furthermore, wherever possible, professional psychological support at future camps to support attendees to manage anxiety should be available. It is also important to consider a camp intervention as a flagship offering within a comprehensive suite of options, allowing individuals to engage on their own terms. Once a young person engages with one option for support, they may feel confident enough to try something more challenging. Consultation with young members of the community is needed to ascertain what these options would include, however, effective examples from the international cleft community currently include podcast episodes,^54^ online groups, councils, and activity day events for young people.^29^

### Implications for Future Research

The mixed methods approach used here allowed for insights that would not have been discernible from the quantitative data alone, a notion supported in previous research.^34,36^ In particular, this builds on previous anecdotal reports of observed change that selected measures have been insensitive to,^36^ or where camps have taken place but evaluative data have not been routinely collected.^37^ It is recommended that future camps and youth events consider evaluation to be a key part of the process and employ a mixed-methods approach to demonstrate multifaceted effects.

Utilizing standardized measures to evaluate such interventions allows for comparison to general population norms and prior studies. The Rosenburg Self Esteem Scale^35^ and Harter Sociability Scale (SPP-Ad)^43^ are psychometrically valid and have been previously used within craniofacial studies^34,46^ and the wider literature. These measures therefore hold utility as screening tools to identify young people who may benefit the most from a camp intervention and in demonstrating whether peer support interventions are effective. The Norwegian Friendship Scale was also useful in demonstrating a tangible impact on participants’ self-perceived quality of friendships, a known buffer for negative social interactions common in cleft and other visible conditions.^11–14,16,55^

There are limitations to the current study which should be acknowledged. First, the BILD-Q provided little additional insight within this study. At all 3 timepoints, the study cohort reported scores above the general population norm, and no statistically significant changes were observed. Therefore, the current data would suggest that broader body image concerns did not significantly impact on life engagement within our study. The BILD-Q measure was originally developed for use in people who may experience weight stigma and may not be sensitive enough to capture the more specific impacts of appearance concerns in young people with visible differences. Similarly, the generic Body Esteem Scale also failed to identify appearance concerns in prior studies,^5,16^ suggesting a cleft-specific measure may be more effective. Therefore, it is recommended that future studies include both general population and cleft/craniofacial-specific measures. Alternatively, it could be that camp was not a powerful enough intervention to significantly impact overall life engagement and/or that the follow-up period was not long enough to assess these changes. Consequently, multiple and longer-term follow-ups after interventions such as camp are recommended.

Another limitation was the use of the SPP-Ad, an outcome measure intended and normed for use in adults, with an adolescent population. This measure was selected as it has been used previously in New Zealand^45^ (and United Kingdom)^46^ cleft studies and since consistent use of outcome measures across studies would allow for future comparison across groups. However, utilizing an adolescent-specific measure may offer a more reliable comparison to general population data and should be considered for future evaluation of adolescent interventions.

Another limitation of this study, as noted in prior literature,^34,38^ is the lack of a direct control group with whom a comparison can be offered. While data could have been collected from a cleft and/or noncleft group who did not receive the intervention, it was anticipated that engagement would have been low. Instead, this study opted to utilize available general population normative data. Although this data offered important insight, it was predominantly collected from North American cohorts which may not be fully comparable to NZ youth. The use of normative data collected in the country under study would have been more robust had it been available and is an important future consideration for similar studies. Furthermore, when interpreting the qualitative data in the absence of a direct control group, it is difficult to discern which concerns are specific to the cleft population versus which concerns may be common to all youth.

Due to the sample size (n* *= 24), generalizability of the findings is limited. Some of the above- and below-norm stratified samples consist of very small sample sizes (eg, n* *= 4), further limiting generalizability. Ongoing evaluation of camp events is warranted to further understand experiences of people who attend interventions such as camp. Additionally, the small sample size precluded analysis by demographic variables; it would be of interest to understand which demographic factors (if any) influence people's camp experience. Finally, the research team could not control for any extraneous environmental factors which may have occurred between the end of camp and follow-up timepoint (eg, returning to school and receiving further cleft-related treatment).

## Conclusion

This mixed methods study of a 3-day, 2-night camp intervention for young people with cleft explored how participation influenced self-reported friendship quality, sociability, life engagement, and self-esteem. Results suggest that attending camp had little effect on the quantitative scores of those already reporting high levels of adjustment, but improvements were observed for those who reported lower levels of sociability and self-esteem at baseline. Qualitative data indicated improvements in self-confidence, reduced feelings of isolation, and a better understanding of cleft for almost all participants, regardless of their scores on standardized measures. The findings of the present study align with evaluations of previous camps for young people with health conditions and lend further support to the value of peer support interventions in cleft and the wider field. It is suggested that support organizations, healthcare teams, and research teams’ work together to deliver camps as part of a wider youth program.
